# Exploring Perceived Changes to Mental Health When Restricting and Resuming Specific Adaptive Daily Actions: Longitudinal Qualitative Substudy Within a Randomized Controlled Trial

**DOI:** 10.2196/82986

**Published:** 2026-01-13

**Authors:** Alana Fisher, Blake F Dear, Alison Dagnall, Heather D Hadjistavropoulos, Olav Nielssen, Lauren G Staples, Rony Kayrouz, Nickolai Titov

**Affiliations:** 1 The eCentreClinic, School of Psychological Sciences, Macquarie University, Level 2 Australian Hearing Hub, 16 University Ave, Macquarie Park, 2113, Australia, 61 0404629858; 2 MindSpot, Macquarie University Health, Macquarie Park, 2113, Australia; 3 Department of Psychology, University of Regina, Regina, SK, Canada

**Keywords:** activities of daily living, coping strategies, goals, habits, health behavior, mental health, qualitative research, self-management, social support

## Abstract

**Background:**

Anxiety and depressive disorders are common and burdensome, yet many people prefer to self-manage and do not access treatment or fail to achieve meaningful improvement. Prior research indicates that the frequency of performing simple, everyday actions, namely “The Things You Do” (TYD; ie, healthy thinking, meaningful activities, having goals and plans, healthy routines, and social connection), is strongly associated with support mental health and well-being. This research has been primarily quantitative in nature, and so less is known about how people perceive and interpret changes in their mental health when engaging in or limiting these actions.

**Objective:**

This study aims to explore participants’ perceptions of mental health changes and associated insights into what most impacts their mental health, during a randomized controlled trial involving the systematic restriction and followed by the resumption of the TYD actions.

**Methods:**

This longitudinal qualitative substudy analyzed weekly free-text comments from 70 healthy Australian adults (intervention group [IG] n=36; control group [CG] n=34). IG participants completed an 8-week randomized controlled trial comprising 3 phases: a 2-week baseline phase (Phase A), a 2-week behavior restriction phase during which they reduced the frequency of the TYD actions (Phase B), and a 4-week recovery or resumption phase during which they increased the frequency of the TYD actions back to usual levels (Phase C). CG participants were instructed to maintain usual habits and activities. The weekly free-text comments were related to what participants had noticed and learned about their mental health. These were thematically analyzed using framework methods to identify patterns in perceived changes, considering trial phase, group allocation, and participant characteristics.

**Results:**

Analyses identified five interrelated themes around what participants reportedly learned and what most impacted their mental health: (1) rhythms of daily life and routine, (2) harnessing internal psychological resources, (3) social support and interpersonal stressors, (4) staying active and enjoying yourself, and (5) environmental and external influences. In the IG, participants reported that behavioral restriction led to subjective disruptions across all 5 themes, precipitating declines in mood, energy, and stability; resumption fostered recovery, along with increased insights into oneself and mental health, coping strategies, and a sense of agency. Compared to the IG, the CG more often emphasized environmental and external influences.

**Conclusions:**

The findings reinforce the centrality of specific daily actions, namely the TYD, to people’s subjective well-being and suggest an additional “macro-level” comprising environmental and external influences. Exposure to behavioral restriction and resumption/recovery served to highlight the importance of certain factors for mental health and appeared to improve people’s sense of agency and locus of control regarding their mental health.

## Introduction

Mental health disorders impose a significant and increasingly heavy burden of disease globally, with anxiety and depressive disorders being the most prevalent [1]. While a range of psychological interventions are available and effective, many people experiencing these symptoms are unable to or choose not to access treatment or do not experience meaningful improvement [2,3]. Accordingly, there has been growing interest in the role and impacts of low-intensity behavioral strategies as more accessible ways to support mental health and well-being. These strategies might include engaging in enjoyable or meaningful activities, maintaining daily routines, and fostering social connection [4-6].

Recent work by Titov and colleagues [7-10] has sought to identify the specific behavioral actions most strongly associated with reduced symptoms of depression and anxiety. This program of research has culminated in the identification of the “Things You Do” (TYD) actions, which are (1) healthy thinking (ie, maintaining realistic, balanced thoughts about yourself, others, and the future), (2) meaningful activities (ie, doing things that bring a sense of accomplishment, satisfaction, or joy), (3) having goals and plans (ie, having something to look forward to, and working toward personal goals), (4) healthy routines (ie, maintaining regular habits like sleep, healthy eating, and physical activity), and (5) social connection (ie, having regular, meaningful contact with people you care about). A series of cross-sectional, longitudinal, and experimental studies have shown that increased engagement with these actions predicts improvements in mental health outcomes, whereas reductions in these behaviors are associated with increased symptoms [7-10]. Additionally, a recent to-be-published randomized controlled trial (RCT; ACTRN12624001491550) demonstrated that systematically restricting then resuming the TYD actions led to an initial induction and a subsequent recovery of depression and anxiety symptoms in a community sample of healthy adults (n=70).

While these findings provide strong empirical support for the relevance of TYD actions to mental health, all studies to date have been quantitative in nature. Less is known about how people perceive and interpret changes in their mental health when engaging in or limiting specific adaptive actions over time, that is, longitudinally. Existing qualitative and mixed methods research using a longitudinal design has provided some relevant and important insights into people’s subjective experience of changes in their mental health over time. These insights include people’s awareness of changes in their mental health in response to life-changing health events (ie, depression in stroke survivors) [11] or treatment initiation (ie, ketamine for treatment-resistant depression) [12] or discontinuation (ie, antidepressant withdrawal) [13], as well as the complex meanings that people attribute to changes in their mental health beyond symptom changes alone, in terms of their identity, agency, and sense of control [11-14]. Additionally, existing research highlights the importance of longitudinal, qualitative mental health assessments to explore people’s subjective experience in a more nuanced way and to mitigate difficulties in retrospective recall and inaccurate reporting [11,12,14,15]. Gaining an in-depth understanding of people’s perceptions of TYD-related changes in their mental health, alongside any learnings gained during this process, can offer important insights into the acceptability, perceived mechanisms, and personal relevance of such interventions.

To address this knowledge gap in our understanding, this study aimed to explore participant views and experiences of mental health changes going through periods of reducing and increasing specific daily activities related to mental health. It is a substudy of the aforementioned RCT (ACTRN12624001491550). Specifically, the research questions guiding this study were as follows: (1) What do participants attribute any perceived changes in their mental health to during the trial? (2) What do participants report learning in response to any perceived changes? (3) To what extent, if any, are these perceived changes influenced by participant group, trial phase, and background characteristics (eg, age, gender, education, baseline symptoms of anxiety and depression, prior help-seeking for mental health)?

In answering these questions, this study sought to complement and build upon the existing quantitative findings on the TYD and mental health. To this end, this study sought to provide a comprehensive understanding of behavioral approaches to mental health, to help inform the design and delivery of the TYD and other similar low-intensity or self-guided interventions.

## Methods

### Study Design

This longitudinal qualitative substudy formed part of an RCT described elsewhere (ACTRN12624001491550), which evaluated the mental health impact of changes in specific daily actions linked to psychological health and well-being (ie, the aforementioned TYD) [9]. The trial employed a 2-arm, parallel-group design with participants randomly stratified by gender, age, and symptom severity and randomly allocated to either an intervention group (IG) or control group (CG). Allocation was performed by a researcher independent of recruitment (BFD), using concealed randomization until eligibility was confirmed during a structured telephone interview.

The IG followed an 8-week protocol comprising three phases, consistent with prior methodology [9]: a 2-week baseline phase (Phase A), a 2-week behavior restriction phase during which the IG was instructed to reduce the frequency of the TYD actions (Phase B), and a 4-week recovery or resumption phase during which the IG was instructed to increase the frequency of the TYD actions back to usual levels (Phase C). Both the IG and the CG completed the same questionnaires at equivalent time points, but the CG was instructed to maintain their usual routines and activities throughout the trial. As expected, the findings from this RCT showed significantly greater increases in symptoms of depression and anxiety for the IG during Phase B compared to the CG, while the scores on all outcome measures were similar for both groups during Phase A and Phase C. In addition, the self-reported ratings of any mental health changes corresponded to changes on the symptom measures.

This qualitative substudy explored participants’ weekly subjective experiences of mental health changes and associated insights during each of the trial phases, using data collected via a purpose-designed questionnaire (see the *Materials and Measures* section).

### Ethical Considerations

Ethics approval for all aspects of the research was obtained from Macquarie University Human Research Ethics Committee (Ref: 520241769358699), and the trial was prospectively registered on the Australian and New Zealand Clinical Trials Registry (ACTRN12624001491550). All participants provided informed consent prior to enrollment and were informed of the behavioral restriction component of the IG. Participants in the IG were also asked to confirm ongoing consent on a weekly basis. All participants provided informed written consent prior to enrollment and were informed of the behavioral restriction component of the IG, along with their right to withdraw at any time without consequence. Participants in the IG were also asked to confirm ongoing consent on a weekly basis. The privacy and confidentiality of participants were maintained throughout the study. In recognition of their time and effort, participants were offered gift vouchers valued at AU $25 (US $16.75) for completing the questionnaires on time each week, and an additional gift voucher valued at AU $50 (US $33.50) for completing the final set of questionnaires. As such, participants could be remunerated a total of AU $250 (US $167.54) in gift vouchers across the course of the trial. For more information, see the registered trial protocol (ACTRN12624001491550).

### Participants and Setting

Participants were recruited nationally through the eCentreClinic website and social media posts (Facebook and X) and via an external recruitment agency (“Build Clinical”). The eCentreClinic is a digital mental health research clinic specializing in clinical trials across a range of mental health and physical health conditions.

The inclusion criteria for both this study and the overall trial were as follows: (1) aged 18 years or older, (2) self-reported symptoms of depression (Patient Health Questionnaire-9 item, PHQ-9 <10) [16] and anxiety (Generalized Anxiety Disorder questionnaire-7 item, GAD-7 <8) [17] in the healthy or mild range, and (3) resident of Australia. The exclusion criteria included inability to read and understand English and current engagement in psychological treatment.

### Procedure

Interested individuals completed an online screening questionnaire and were invited to a structured telephone interview with a registered mental health professional (NT or AD) to confirm eligibility and review study procedures. During this call, NT or AD explained the behavioral restriction phase (Phase B) to all participants. IG participants were provided with a “Restriction Guide” and received a follow-up call before or at the start of the restriction phase to go through this guide, and confirm the start date, 2-week duration, trial purpose, and address any questions. In addition, upon starting the restriction phase, a reminder email was sent to IG participants.

Of the 103 applicants between February 3‐21, 2025, 70 met all inclusion criteria and were randomized to the IG and the CG. All participants were monitored by NT and AD for safety and risk over the course of the trial. IG participants received weekly brief phone calls (<5 min) from week 2 onward, while CG participants were only called if there were clinical concerns or to prompt questionnaire completion. These calls followed a script and were used to confirm participants’ understanding of the current trial phase (eg, restriction or recovery), reiterate key instructions for the phase, conduct a brief well-being check, and answer any questions. Participants were also reminded of how to contact the research team if needed. During these calls, NT and AD took notes of their discussion with participants, but no specific therapeutic or mental health support was offered. These notes aimed to capture information about additional comments expressed by participants in the weekly call, as well as clinical observations of those calls. Throughout the recovery phase of the trial (Phase C), IG participants were sent 1 brief SMS message each weekday (for the full 4 wk) encouraging them to do a target TYD action (eg, for healthy routines: “Healthy habits start with a good routine. Plan out your day so that you can get things done, but also have time for what makes you feel good. Routines matter”).

### Materials and Measures

This study draws on a subset of measures included in the overall trial. All included measures are described in the registered trial protocol (ACTRN12624001491550).

#### Reflections Questionnaire

The purpose-designed reflections questionnaire (RQ) was administered weekly from weeks 2 to 8, and again at posttrial (also week 8). It consisted of three items: (1) a Likert-style question assessing perceived change in mental health in the past week (from “greatly improved” to “greatly deteriorated,” with “no change or normal” at the midpoint), and 2 open-ended items asking participants to (2) describe the reasons for any changes, and (3) reflect on what they had learned about their mental health. In the final administration of the questionnaire, an additional question was included with slight rewording asking participants to reflect on perceived changes and insights from across the whole trial.

#### Sociodemographic and Clinical Characteristics

To characterize the participant sample and their mental health trajectory during the trial and explore potential links between their responses on the RQ and background characteristics, the following information was also included: age, gender, educational attainment, symptoms of depression (PHQ-9) [16] and anxiety (GAD-7) [17], past mental health seeking (if so), and type of health professional seen (eg, general practitioner psychologist). With regard to the PHQ-9 and the GAD-7, higher scores correspond to more severe depression and anxiety symptomatology, respectively. Of relevance to this study of healthy adults, scores of 0‐4 indicate minimal or no symptoms whereas scores of 5‐9 indicate mild symptoms.

### Analysis

This substudy focuses on participants’ free-text responses to the open-ended items on the RQ (ie, items 2 and 3), collected weekly and at posttrial. All analyses were conducted by the lead author, AF, a postdoctoral researcher with a background in applied clinical or health psychology and expertise in qualitative research in mental health populations. All responses were analyzed thematically using framework methods [18]. This analytic approach allowed the research team to discern common patterns (subthemes or themes) in the responses of participants both within individuals across the phases of the trial (longitudinally) as well as between individuals in specific phases of the trial (cross-sectionally). Additionally, analyses sought to explore the potential qualitative links between participant responses, trial phase (Phase A, B, C, posttrial), group allocation (IG vs. CG), and background characteristics (eg, age, past help-seeking).

AF developed a set of initial codes along with a preliminary map of higher order subthemes or themes based on the responses from a randomly selected subsample of 20% (n=14) of the participants across all phases of the trial. These subthemes or themes were then discussed and refined to minimize duplication or repetition and to ensure that they were comprehensive in their coverage, sufficiently distinct, and named in a way that accurately reflected their content. AF led this process in collaboration with the other members of the multidisciplinary research team whose backgrounds included psychology, psychiatry, digital mental health, and qualitative or mixed-methods research (eg, NT, AD, LGS, BFD, ON, HDH). AF then coded all participant responses with the assistance of the NVivo software (version 15; QSR International), meeting with the team regularly to continue iterating and refining the thematic map. To explore any potential relationships between participant responses, trial phase, and group allocation, separate files were created for each phase of the trial. This meant that each participant had up to 4 files, ie, for Phase A, B, C, and posttrial. Only files containing codable data were included; files with responses such as “N/A,” “no change,” “nothing,” and so forth were excluded from analyses.

Analyses were mainly inductive (data driven) and took a postpositivist approach, such that participant responses were taken at face value and as describing reality, with minimal interpretation [16]. This approach was chosen because participants had recorded their responses remotely and without the opportunity for a researcher to probe for more contextual detail or clarification. Once all participant responses were coded in NVivo, they were exported to a framework matrix created in Microsoft Excel [17]. At this point, AF noted any marked differences in the relative prevalence or frequency of the codes or subthemes or themes based on participant group allocation, trial phase, or background characteristics. Lastly, AF read over and synthesized the weekly clinician notes (by NT and AD), triangulating these with the identified codes or subthemes or themes and chose illustrative participants' quotes for each of the subthemes.

## Results

### Participant Characteristics

Participants’ baseline sociodemographic and clinical characteristics will be described in a separate, forthcoming publication. Broadly speaking, the sample was aged on average in their mid-thirties (CG: mean 34.7, SD 13.2 y; IG: mean 37.4, SD 9.4 y) and predominantly female (CG: n=24, 68.6%; IG: n=26, 78.8%), with university level education (CG: n=21, 60%; IG: n=31, 93.9%). A minority of the sample had previously consulted a mental health professional (eg, GP, psychologist, and/or counselor) (CG: n=5, 14.3%, IG: n=4, 12.1%) and indicated very mild-to-mild difficulties with anxiety (CG: n=6, 17.2%; IG: n=8, 24.3%). No one indicated any difficulties with depression. There were no significant between-group differences, except for the level of education, with a significantly higher proportion of the IG having university-level education compared to the CG.

During the course of the trial, the average PHQ-9 (depression) scores ranged as follows: for Phase A weeks 1 and 2 (IG: mean 1.5‐1.5, SD 1.4‐1.6; CG: mean 2.1‐2.3, SD 2.8‐2.9), Phase B weeks 3 and 4 (IG: mean 1.8‐7.0, SD 2.9‐4.6; CG: mean 1.7‐1.7, SD 2.3‐2.6), and Phase C weeks 5-8 (IG: mean 1.2‐6.9, SD 1.3‐3.4; CG: mean 0.9‐1.3, SD 1.4‐1.9). Meanwhile, the average GAD-7 (anxiety) scores ranged as follows: for Phase A weeks 1 and 2 (IG: mean 1.1‐1.2, SD 1.5‐1.6; CG: mean 1.2‐1.4, SD 1.6‐2.1), Phase B weeks 3 and 4 (IG: mean 5.6‐5.9, SD 3.0‐4.3; CG: mean 1.1‐1.2, SD 1.7‐2.3), and Phase C weeks 5-8 (IG: mean 0.9‐5.6, SD 1.6‐3.0; CG: mean 0.9‐1.0, SD 1.4‐1.7).

Attrition was low for the parent RCT and therefore this substudy: 1 IG participant and 1 CG participant did not start, while a second IG participant withdrew in week 2 (Phase A) due to unrelated physical illness. In this substudy, a total of 231 participant files were included for qualitative analyses with similar numbers across each phase and group of the trial (Phase A: n=25 IG, n=25 CG; Phase B: n=32 IG, n=28 CG; Phase C: n=31 IG, n=31 CG; posttrial: n=30 IG, n=29 CG).

The thematic analysis identified 5 overarching themes about what participants perceived impacted their mental health, each comprising several subthemes: all of which mapped across all 4 phases of the trial and both participant groups. The themes appeared to be to some extent overlapping and interrelated; for example, when the rhythms of daily life and routine (Theme 1), social support and interpersonal stressors (Theme 3), and staying active and enjoying oneself (Theme 4) were all functioning well, these served to support one’s internal psychological resources (Theme 2), while buffering against environmental and external influences (Theme 5). By contrast, when Themes 1-3 were disrupted (as was the case for the IG during the behavioral restriction phase, Phase B), they served to challenge one’s internal psychological resources (Theme 2) and render participants more vulnerable to the negative impacts of environmental and external influences (Theme 5). As the CG was not exposed to behavioral restriction and resumption or recovery (in Phases B and C), the importance of Themes 1, 3, and 4 appeared to be less salient to them, and they had fewer opportunities to challenge and harness their internal psychological resources (Theme 2), while also more frequently describing the environmental and external influences (Theme 5) on their mental health compared to the IG.

In instances where the relative prevalence or salience of a particular theme or subtheme appears to differ according to trial phase, participant group, or background characteristics, this is noted in the following sections. Illustrative quotes for each of the subthemes or themes are provided in Table 1.

**Table 1.** Illustrative participant quotes organized by theme and subtheme.

Theme^a^	Subtheme	Illustrative participant quote
Theme 1: Rhythms of daily life and routine	Keeping a consistent “healthy” routine is key Organization and scheduling to “stay on track” Sleep as a pillar for mental health Physiological and hormonal fluctuations	“Changing my healthy habits...I learnt that my mental health can be improved by doing healthy habits and it has been affected by stopping these habits or restricting them.” (ID29_PhaseB_Intervention) “I have been a little more organized this week by noting down appointments, assessments, classes, work and calls to keep on track and not be surprised.” (ID62_PhaseB_Control) “Sleep…keeps me happy and improves my mental health...not being able to do that has a ripple effect on me. I get easily irritated and angry and I go in my shell...It also impacts my energy levels.” (ID17_PhaseB_Intervention) “I find that my mental health sometimes changes with my menstrual cycle.” (ID32_PhaseA_Intervention)
Theme 2: Harnessing internal psychological resources	Cognition-focused strategies for self-regulation Emotion-focused strategies for self-regulation Building self-awareness and insight into mental health	“I am in control of my mental health ie, in the way I choose to frame situations and the way I choose to react to good and bad news.” (ID20_PhaseA_Intervention) “That being kind to myself is important and that to not put pressure on myself and to just do the best I can.” (ID47_PhaseC_Control) “...the disruption period was hard for me to climb out of. [It] Has taken longer time than I thought [it would]...my mental health is more fragile than I once thought...”(I D5_PhasePosttrial_Intervention)
Theme 3: Social support and interpersonal stressors	Social support and connection for mental health Interpersonal stressors affecting mental health	“Catching up with friends has helped boost my mental health. Being around positive people has helped boost my happiness.” (ID13_PhaseC_Intervention) “[I’m] Feeling overwhelmed by juggling kids home on school holidays and trying to get work done in amongst it all while still trying to create memories for them and facilitate fun activities for them.” (ID34_PhaseC_Intervention)
Theme 4: Staying active and enjoying yourself	Engaging in enjoyable and purposeful activities Role of (physical) activity in regulating mood and anxiety	“I think having a strong sense of purpose and feeling like I’m working towards the achievement of that is really important to my sense of wellbeing.” (ID33_PhaseC_Intervention) “Exercising is important to me beyond just the act itself as it regulates my sleep, balances my schedule and improves my mood.” (ID28_PhaseA_Intervention)
Theme 5: Environmental and external influences	External stressors and demands Your environment matters	“I do feel that my mental health changed a little in the last week...primarily due to extra tasks and deadlines at work. I found myself needing to spend a lot more time completing things, and often bringing work home...” (ID32_PhaseC_Intervention) “My house mess reflects my mental health. I just can’t seem to keep up with housework…[it]loses out to other responsibilities.” (ID55_PhaseB_Control)

a Across themes, differences were evident by participant characteristics and group. Themes 1 and 2 were more salient among intervention group participants, particularly during restriction (Phase B) and resumption or recovery (Phase C), and among those reporting baseline anxiety. Themes 3 and 4 were more frequently referenced by younger participants and those without prior help-seeking, with social connection especially salient among female participants. Theme 5 was the only theme more prominent among control group participants, particularly younger and middle-aged adults with baseline anxiety. Gender differences were most apparent in Themes 2 and 3, with female participants describing a broader range of psychological and interpersonal strategies.

### Theme 1: Rhythms of Daily Life and Routine

Theme 1 was highly prevalent within participant accounts (269 coded instances across the 231 participant files) and was present across both groups and all phases of the trial. This theme appeared to be especially salient among IG participants relative to CG participants during the restriction (Phase B) and resumption or recovery phases (Phase C) of the trial. Some but not all subthemes (eg, the importance of routine) within this theme appeared to be more salient among participants with some self-reported baseline anxiety compared to those without.

Many participants reported that maintaining (and in the case of the IG, also reinstating) a consistent “healthy” daily routine was a core strategy for regulating their mood, anxiety, energy, and thinking. Specifically, participants articulated the importance of regular patterns of sleep, nutrition, physical activity, and social interaction. Both the IG and the CG commonly reported disruptions to these patterns; for IG participants, these disruptions were mainly attributed to behavioral restriction (Phase B), while CG participants cited factors such as changes in health or illness, or external obligations (eg, family, work, study). Regardless of the perceived cause, disrupted routines reportedly precipitated emotional lability, a general sense of malaise and instability.

According to participants, sleep was foundational and a “pillar of mental health.” As such, a lack of or “poor” sleep (often in interaction with other factors such as fatigue, illness, and stress) quickly led to worse mood and anxiety, while “good” sleep had the opposite effect. For IG participants, sleep appeared to be a primary driver of both worsened and improved mental health across the restriction (Phase B) and resumption or recovery phases (Phase C) of the trial.

Fewer participants cited organization and scheduling as helping them to “stay on track” and balance various life demands (eg, work, study, family commitments). Participants also cited the importance of being flexible and needing to accommodate and adapt to change; indeed, breaks from routine were also seen as having a positive effect on their mental health when taken in the form of holidays, days off work, and long weekends. In addition, a few participants (all women) reported that hormonal and physiological fluctuations, such as menstruation, (peri)menopause, and migraines, influenced their mood by way of disrupting their usual routine (eg, sleep, physical activity).

### Theme 2: Harnessing Internal Psychological Resources

As with Theme 1, Theme 2 was also highly prevalent within the sample (278 coded instances across the 231 participant files) and was identified in both groups and across all phases of the trial. Participants, especially female participants, described a repertoire of psychological strategies to help regulate their emotional responses and navigate stressors. Participants alluded to both cognition-based and emotion-based strategies; cognition-based strategies included cognitive flexibility (reappraisal and reframing) and balancing tolerance of uncertainty with realistic expectations around their personal sense of control and influence over others. Emotion-based strategies included maintaining a positive mindset and “self-talk,” along with self-compassion and taking time to emotionally remove oneself and reflect rather than react with heightened emotion.

These strategies, in particular cognitive-based strategies, were especially salient among IG participants relative to CG participants during the behavioral resumption or recovery phase of the trial (Phase C). For all participants, these strategies reportedly buffered against psychological distress and enhanced their emotional resilience.

Participants, especially female participants and those in the IG during the behavioral resumption or recovery phase (Phase C), described building self-awareness and insight into the “ups and downs” or “fragility” of their mental health, identifying personal triggers, and developing bespoke coping “toolkits,” which incorporated a combination of personally relevant and useful strategies (eg, positive mindset, reframing). These participants articulated a growing sense of agency and accountability in managing their well-being, which included making a concerted effort to do more of the things known to positively impact their mental health, less of those known to negatively impact their mental health, and committing to process over outcomes.

### Theme 3: Social Support and Interpersonal Stressors

Theme 3 appeared to be less prevalent within participant accounts compared to the first two (91 coded instances across the 231 participant files). That said, Theme 3 was still present across both groups and all phases of the trial, with greater predominance among female compared to male participants and younger or general age adults compared to older adults (aged 55 y and over).

Socializing and connection with loved ones (eg, friends, family, partner) as well as “positive” others (eg, colleagues, new friends, a pet) appeared to be key determinants of emotional well-being. These interactions, and for some, particularly in-person interactions, were described as vital sources of support, belonging, validation, and emotional “grounding.” By contrast, negative or challenging relationship dynamics or interactions, including via social media, were associated with emotional dysregulation, heightened anxiety, and loneliness.

The bidirectional link between social withdrawal versus connection and negative versus positive mental health was especially evident in the accounts of IG participants during the behavioral restriction (Phase B) and the resumption or recovery phases of the trial (Phase C). Across both groups and all phases, a few participants also felt that heightened family demands affected their mental health, with caregiving roles especially taking an emotional toll (eg, young children, older adults, or sick parents).

### Theme 4: Staying Active and Enjoying Yourself

As with Theme 3, Theme 4 was also less prevalent within participant accounts (109 coded instances across the 231 participant files), although still present across both groups and all phases of the trial. Participants, especially those reporting no prior help-seeking with a mental health professional, consistently emphasized the importance of staying (physically) active and engaging in activities that were enjoyable, purposeful, and meaningful. The importance of these activities appeared to be more salient in the accounts of IG participants than CG participants during the behavioral restriction phase (Phase B). Physical activity, both in the form of structured exercise and leisure-based movement (eg, walking, hiking), was described not only as mentally and physically energizing but also as central to mood regulation and a sense of well-being.

Participants also expressed that staying “purposefully busy” and pursuing personal goals, whether through work, volunteering, or personal projects, served as a positive distraction and helped to ease worry. Conversely, excessive passive activities, such as screen time or “doom-scrolling” (ie, mindlessly viewing high volumes of social media content without intending to), were seen as having a negative impact on mental health, and participants sought to limit these.

### Theme 5: Environmental and External Influences

Theme 5 was the least prevalent within participant accounts (64 coded instances across the 231 participant files), although still present across both groups and all phases of the trial. This theme appeared to be more frequently described by younger and middle-aged adults (ie, younger than 45 years old), those reporting some anxiety at baseline, and those without any prior help-seeking with a mental health professional. This theme was also the only one to be more salient within the accounts of CG participants compared to IG participants. Accordingly, there did not appear to be any differences in the prevalence of this theme across the trial phases.

Within this theme, external stressors, such as work and study pressures, financial concerns, and pending major life decisions, had a negative impact on mental health. These stressors reportedly impacted daily rhythms and routines, led to emotional lability, and triggered maladaptive coping responses such as withdrawing from family and friends or working longer hours. By contrast, several participants reported that resolving or gaining clarity on major life decisions (eg, purchasing a house) or stressors (eg, a family member’s diagnosis) led to improvements in their mood and anxiety.

In addition, one’s immediate surrounding environment was described as a key influence on mental health. Here, participants alluded to both their physical environment as well as their experiential environment. For example, chaotic or, by contrast, calm home environments shaped and were shaped by a negative or positive mental state, while access to personal “mental” space and time outdoors and in nature was described as calming and restorative. Weather and seasonal changes also reportedly had an influence on affect for a few participants, sometimes but not always by way of changes in their behavior (eg, sleep, exercise, diet).

### Weekly Clinician Notes—Confirmatory Insights Into IG Participants’ Mental Health Trajectory

A review of the weekly clinician notes confirmed the above-described themes and subthemes, while also providing some additional insights into the mental health trajectory of participants in the IG. The notes revealed that, even at baseline (Phase A), the IG participants anticipated that breaking their usual routines would be challenging and detrimental to their mental health, which was realized during the behavioral restriction phase (Phase B). The impact of these breaks in routine on mood and anxiety appeared to compound over time, with the IG participants reporting increasing fatigue and emotional discomfort. That said, it was also during the behavioral restriction phase (Phase B) that some participants started developing early insight into the importance of routine for their well-being.

When starting the behavioral resumption or recovery phase (Phase C), most IG participants experienced gradual improvements in their mental health with a return to their routine, albeit with some lingering anxiety and low mood. During this phase, IG participants felt that the SMS reminders acted as a supportive mental health aide. While the pace of recovery varied, by the end of the trial, participants across both groups expressed greater self-awareness, highlighted personal learnings, and evaluated the trial experience positively.

## Discussion

### Overview

This study provides in-depth qualitative insights into how individuals perceive and make sense of mental health changes when engaging in or limiting simple, daily actions (ie, the “TYD”). These findings not only reinforce the importance of these behaviors to people’s emotional well-being, but they also reveal the context-dependent ways in which people interpret changes to their mental health and develop strategies and self-awareness in response to behavioral restriction and resumption or recovery.

### Principal Findings and Comparison With the Broader Literature

Based on the current findings, healthy adults tend to attribute perceived changes in their mental health most frequently to shifts in their daily routines and use of psychological coping strategies, followed by engagement in enjoyable and purposeful activities, the availability and quality of social support, and exposure to environmental and external stressors. Despite our sample having received no specific psychoeducation in the “TYD” until toward the end of the trial, the identified themes broadly map onto the 5 domains of healthy routines, healthy thinking, meaningful activities, goals and plans, and social connections, with the addition of responding and adapting to one’s environment. This additional theme may be subsumed by the existing TYD actions, for example, one may adapt to their environment by way of healthy thinking (eg, developing realistic thoughts about the world) or social connections (eg, being part of a community) [7,9]. Alternatively, this additional theme may represent an expansion to the TYD model, in so far as environmental and external influences operate at a “macro” level and shape one’s capacity to engage at the “micro” level, that is, regularly practice actions to improve mental health. This integration of “macro” and “micro” elements within mental health is consistent with current research focusing on the broader social and environmental determinants of psychological distress and well-being [19,20]. Also building on prior research into adaptive actions for mental health, some themes were more frequently cited within our sample, suggesting that, for healthy adults, some action domains (eg, healthy routines, healthy thinking) are seen to more strongly influence their mental health than others (eg, social connections). Future research would need to test this hypothesis in a larger, more representative community sample, as well as in adults with clinically significant mental health symptoms.

Behavioral restriction (ie, Phase B for the IG) often led to disruptions in sleep, physical activity, and social contact, precipitating declines in mood, energy, and emotional stability. Meanwhile, behavioral resumption or recovery (ie, Phase C for the IG) led to gradual improvements mostly by way of reinstating healthy daily routines and activities, which some credited to receiving the daily SMS prompts and weekly questionnaires. In the absence of any structured restriction or recovery (ie, Phases B and C for the CG), external pressures, such as work, study, finances, and home environments, appear to exert a greater influence on people’s mental health. These findings suggest possible causal mechanisms between withdrawing or disengaging from certain daily activities and subsequent deteriorations in mental health. One such mechanism of key importance appears to be disrupted sleep and its resulting negative impacts on energy levels, activity, and fatigue. Aligning with our findings, other research has confirmed sleep’s role as a “pillar of mental health,” with positive correlations between sleep quality and mental health in the general adult population [21,22] and RCT interventions designed to improve sleep quality, also leading to improved mental health [23]. Future research would need to test this within a causal model. However, the current findings suggest that prioritizing regular sleep-wake patterns in times of significant disruptions or stressors (eg, routine changes due to illness, starting a new job, moving cities or towns, or having a baby) may help to maintain good mental health.

Based on our findings, even a relatively short period of behavioral restriction (two weeks) led to the IG gaining greater self-awareness of the factors shaping their mental health and well-being, identifying personal triggers and buffers, and making use of bespoke coping “toolkits” incorporating emotion-focused (eg, self-compassion, positive self-talk) and more often, cognitive strategies (eg, reframing, maintaining realistic expectations). Research in Western cultures has shown that cognitive strategies, such as cognitive reappraisal or reframing, promote emotion regulation and are generally more adaptive than some emotion-focused strategies, such as expressive suppression [24,25]. It is noteworthy also that these insights into one’s mental health and coping were not limited to the IG; these insights were also evident, albeit to a lesser extent, in the accounts of CG participants. As a result, simply tracking one’s mental health symptoms and being asked to reflect on any spontaneous changes, triggers, or learnings may be a subjectively useful intervention in and of itself [14]. This would need to be confirmed in a future study, especially considering limited and uncertain evidence on links between symptom monitoring and improvements, and the possible effect on the control arm of any RCTs [26,27].

This said, the IG’s preponderance to attribute mental health changes—both deteriorations and improvements—to their own thinking, behavior, and ways of responding suggests that behavioral restriction and resumption or recovery increased their sense of agency and internal locus of control with regard to mental health. Consistent with this suggestion, the CG was more likely than the IG to cite environmental and external influences as being responsible for their mental health changes, in the absence of any behavioral restriction and resumption or recovery. A greater sense of agency and internal (mental health) locus of control may be 2 serendipitous benefits of the current ultra-light intervention in a group of healthy volunteers, as shown with more targeted psychological and health behavioral interventions [28,29]. In addition, these benefits may carry forward insofar as a more internal health locus of control is associated with less psychological distress, lower prevalence of depression and anxiety, and better health care access and outcomes [28,30]. Future research may consider measuring any specific improvements in agency, locus of control, and cognitive emotion regulation strategies as a result of engaging in the “TYD” or similar mental health promoting actions.

Not only did the prevalence or salience of some themes appear to differ by participant group and trial phase, but there was also some preliminary evidence for the influence of background characteristics. Specifically, women, adults of working or study age (ie, aged in their 20s, 30s, and 40s), those with baseline anxiety symptoms, and those without prior mental health help-seeking reported greater sensitivity to disruptions or stressors. While these findings should be treated with caution due to the relatively small number of mentions for some subthemes or themes, the non-representative sample, and the fact that analyses were exploratory, they suggest that some factors are more important to the mental health of different groups. These findings also suggest that ultralight behavioral interventions such as the “TYD” could be tailored for different groups to be maximally relevant and beneficial (eg, younger vs older adults, healthy individuals vs individuals with moderate-to-severe symptoms, first-time help seekers vs those who have completed psychological treatment). By contrast, maintaining (and for the IG, reinstating) a regular healthy routine and daily habits appeared to be universally recognized as important for mental health. In this way, a healthy routine might be promoted as a universal strategy for good mental health, as supported by research conducted during the COVID-19 pandemic [31,32].

### Strengths and Limitations

The findings of this study need to be considered in light of several strengths and limitations. Strengths include the use of longitudinal, qualitative assessments together with weekly clinician notes to gain in-depth and detailed accounts of perceived mental health changes both over time and in response to behavioral restriction and resumption or recovery. Another strength is the low levels of attrition in the sample, ensuring that the findings accurately capture the mental health trajectories of the full sample rather than just a subsample of highly engaged participants. The limitations of the study include the nonrepresentative nature of this sample of healthy volunteers, which was predominantly female and university educated, and therefore had potentially higher levels of mental health literacy [33] compared to the general adult population. Future research would need to ascertain whether the current findings are transferrable to a larger, more representative sample, comprising more male individuals and people without university-level education. Another limitation of the study is that the free-text comments needed to be analyzed at a face level, with minimal interpretation. This was done because the free-text comments were collected remotely, without the opportunity for prompting for future explanation or contextual detail. Remote data collection may be seen as a strength, insofar as it permitted participants to reflect and respond in a self-paced and spontaneous way and may have made some participants more comfortable to disclose sensitive information. However, future research may wish to include weekly interviews to better understand how people perceive and interpret changes in their mental health.

### Conclusions

This study provides novel qualitative evidence on how healthy adults perceive and interpret changes in their mental health both over time and in response to restricting and resuming specific daily actions related to mental health. The findings reinforce the relevance of the “TYD” actions to both mental health deterioration and improvements, while highlighting the potential role of environmental and external influences as an additional, “macro-level” consideration within the model. Even a brief period of behavioral restriction prompted perceived declines in well-being, often via disruptions to sleep and its associated impacts, but also fostered greater self-awareness, coping strategies, and a sense of agency. Taken together, these findings elucidate potential mechanisms of change and some unintended benefits of ultra-light behavioral interventions, such as the “TYD.” While maintaining a regular routine and daily habits appear to be universally important for people’s mental health, other factors may vary in terms of their perceived impact. This underscores both the broad appeal of the “TYD” as well as opportunities for tailoring to ensure it is as relevant and beneficial as possible to different groups in the population.

## Abbreviations

CG: control group
GAD-7: Generalized Anxiety Disorder questionnaire-7 item
IG: intervention group
PHQ-9: Patient Health Questionnaire-9 item
RCT: randomized controlled trial
RQ: reflections questionnaire
TYD: Things You Do

## References

[1] Fan Y, Fan A, Yang Z, Fan D (2025). Global burden of mental disorders in 204 countries and territories, 1990-2021: results from the global burden of disease study 2021. BMC Psychiatry.

[2] Coombs NC, Meriwether WE, Caringi J, Newcomer SR (2021). Barriers to healthcare access among U.S. adults with mental health challenges: a population-based study. SSM Popul Health.

[3] Kessler RC, Kazdin AE, Aguilar-Gaxiola S (2022). Patterns and correlates of patient-reported helpfulness of treatment for common mental and substance use disorders in the WHO World Mental Health Surveys. World Psychiatry.

[4] Hooker SA, Masters KS, Vagnini KM, Rush CL (2020). Engaging in personally meaningful activities is associated with meaning salience and psychological well-being. J Posit Psychol.

[5] Kazdin AE (2024). Interventions in everyday life to improve mental health and reduce symptoms of psychiatric disorders. Am Psychol.

[6] Martland R, Korman N, Firth J, Stubbs B (2023). The efficacy of exercise interventions for all types of inpatients across mental health settings: a systematic review and meta-analysis of 47 studies. J Sports Sci.

[7] Bisby MA, Jones MP, Staples L, Dear B, Titov N (2024). Measurement of daily actions associated with mental health using the Things You Do Questionnaire-15-item: questionnaire development and validation study. JMIR Form Res.

[8] Bisby M, Staples L, Dear B, Titov N (2024). Changes in the frequency of actions associated with mental health during online treatment: analysis of demographic and clinical factors. JMIR Form Res.

[9] Titov N, Dear BF, Nielssen O, Barrett V, Kayrouz R, Staples LG (2024). A pilot study examining whether restricting and resuming specific actions systematically changes symptoms of depression and anxiety. A series of N-of-1 trials. Behav Res Ther.

[10] Titov N, Dear BF, Bisby MA (2022). Measures of daily activities associated with mental health (Things You Do Questionnaire): development of a preliminary psychometric study and replication study. JMIR Form Res.

[11] Kouwenhoven SE, Kirkevold M, Engedal K, Biong S, Kim HS (2011). The lived experience of stroke survivors with early depressive symptoms: a longitudinal perspective. Int J Qual Stud Health Well-being.

[12] Lascelles K, Marzano L, Brand F, Trueman H, McShane R, Hawton K (2020). Ketamine treatment for individuals with treatment-resistant depression: longitudinal qualitative interview study of patient experiences. BJPsych Open.

[13] Smit AC, Snippe E, Bringmann LF, Hoenders HJR, Wichers M (2023). Transitions in depression: if, how, and when depressive symptoms return during and after discontinuing antidepressants. Qual Life Res.

[14] White KM, Dawe-Lane E, Siddi S (2023). Understanding the subjective experience of long-term remote measurement technology use for symptom tracking in people with depression: multisite longitudinal qualitative analysis. JMIR Hum Factors.

[15] Hermanowicz JC (2013). The longitudinal qualitative interview. Qual Sociol.

[16] Howell KE (2013). An Introduction to the Philosophy of Methodology.

[17] Meyer DZ, Avery LM (2009). Excel as a Qualitative Data Analysis Tool. Field methods.

[18] Ritchie J, Lewis J, Nicholls CM, Ormston R (2013). Qualitative Research Practice: A Guide for Social Science Students and Researchers.

[19] Kirkbride JB, Anglin DM, Colman I (2024). The social determinants of mental health and disorder: evidence, prevention and recommendations. World Psychiatry.

[20] Lawrance EL, Thompson R, Newberry Le Vay J, Page L, Jennings N (2022). The impact of climate change on mental health and emotional wellbeing: a narrative review of current evidence, and its implications. Int Rev Psychiatry.

[21] Freeman D, Sheaves B, Goodwin GM (2017). The effects of improving sleep on mental health (OASIS): a randomised controlled trial with mediation analysis. Lancet Psychiatry.

[22] Milojevich HM, Lukowski AF (2016). Sleep and mental health in undergraduate students with generally healthy sleep habits. PLoS One.

[23] Scott AJ, Webb TL, Martyn-St James M, Rowse G, Weich S (2021). Improving sleep quality leads to better mental health: a meta-analysis of randomised controlled trials. Sleep Med Rev.

[24] Hu T, Zhang D, Wang J, Mistry R, Ran G, Wang X (2014). Relation between emotion regulation and mental health: a meta-analysis review. Psychol Rep.

[25] McRae K (2016). Cognitive emotion regulation: a review of theory and scientific findings. Curr Opin Behav Sci.

[26] Kendrick T, El-Gohary M, Stuart B (2016). Routine use of patient reported outcome measures (PROMs) for improving treatment of common mental health disorders in adults. Cochrane Database Syst Rev.

[27] Wang K, Varma DS, Prosperi M (2018). A systematic review of the effectiveness of mobile apps for monitoring and management of mental health symptoms or disorders. J Psychiatr Res.

[28] Mozafari S, Yang A, Talaei-Khoei J (2024). Health locus of control and medical behavioral interventions: systematic review and recommendations. Interact J Med Res.

[29] Yeager CM, Benight CC (2022). Engagement, predictors, and outcomes of a trauma recovery digital mental health intervention: longitudinal study. JMIR Ment Health.

[30] Kesavayuth D, Poyago-Theotoky J, Tran DB, Zikos V (2020). Locus of control, health and healthcare utilization. Econ Model.

[31] Murray G, Gottlieb J, Swartz HA (2021). Maintaining daily routines to stabilize mood: theory, data, and potential intervention for circadian consequences of COVID-19. Can J Psychiatry.

[32] Pilz LK, Couto Pereira NS, Francisco AP (2022). Effective recommendations towards healthy routines to preserve mental health during the COVID-19 pandemic. Braz J Psychiatry.

[33] Chng J, Burns R, Murray K, Crisp D (2022). A community mental health and wellbeing literacy study among Australian adults. J. Happiness Health.

